# Material Composition Characteristics of *Aspergillus cristatus* under High Salt Stress through LC–MS Metabolomics

**DOI:** 10.3390/molecules29112513

**Published:** 2024-05-26

**Authors:** Luyi Xie, Lihong Zhou, Rongrong Zhang, Hang Zhou, Yi Yang

**Affiliations:** Key Laboratory of Plant Resource Conservation and Germplasm lnnovation in Mountainous Region (Ministry of Education), College of Life Sciences, Institute of Agro-Bioengineering, Guizhou University, Guiyang 550025, China; xly1354626375@outlook.com (L.X.); zhang1718812203@outlook.com (R.Z.); zhouhang2361@outlook.com (H.Z.); yangyi2290767532@outlook.com (Y.Y.)

**Keywords:** *Aspergillus cristatus*, high salt stress, substance metabolism, composition characteristics, adaptation mechanism

## Abstract

*Aspergillus cristatus* is a crucial edible fungus used in tea fermentation. In the industrial fermentation process, the fungus experiences a low to high osmotic pressure environment. To explore the law of material metabolism changes during osmotic pressure changes, NaCl was used here to construct different osmotic pressure environments. Liquid chromatography–mass spectrometry (LC–MS) combined with multivariate analysis was performed to analyze the distribution and composition of *A. cristatus* under different salt concentrations. At the same time, the in vitro antioxidant activity was evaluated. The LC–MS metabolomics analysis revealed significant differences between three *A. cristatus* mycelium samples grown on media with and without NaCl concentrations of 8% and 18%. The contents of gibberellin A3, A124, and prostaglandin A2 related to mycelial growth and those of arabitol and fructose-1,6-diphosphate related to osmotic pressure regulation were significantly reduced at high NaCl concentrations. The biosynthesis of energy-related pantothenol and pantothenic acid and antagonism-related fluvastatin, aflatoxin, and alternariol significantly increased at high NaCl concentrations. Several antioxidant capacities of *A. cristatus* mycelia were directly related to osmotic pressure and exhibited a significant downward trend with an increase in environmental osmotic pressure. The aforementioned results indicate that *A. cristatus* adapts to changes in salt concentration by adjusting their metabolite synthesis. At the same time, a unique set of strategies was developed to cope with high salt stress, including growth restriction, osmotic pressure balance, oxidative stress response, antioxidant defense, and survival competition.

## 1. Introduction

Most fungi survive under good physiological conditions, but under high-salt environmental conditions, during long-term adaptive evolution, fungi develop some special physiological structures and produce some special metabolites to survive.

Under high salt conditions, fungi chiefly regulate the osmotic balance by accumulating compatible solutes [1,2,3,4,5], such as sugars, polyols, and amino acids. *Aspergillus sydowii* [6] cultured in a 5.13 M NaCl environment produced trehalose, mannitol, arabitol, and erythritol during growth. Among them, mannitol and arabitol were only synthesized in a saturated NaCl medium. The amount of these substances decreased as the strain growth time was prolonged, whereas the glycerol content remained unchanged throughout growth. When *A. montevidensis* [7] was grown in a high-salt environment, the fungal cells extensively accumulated amino acids, soluble sugars, saturated fatty acids, and other carbon and nitrogen compounds, such as neohesperidin, biuret, aspartic acid, and alanine, as osmotic regulators. These regulators helped the fungi adapt to the high-salt environment and grow. In a high-salt environment, *A. ruber* [8] was induced to synthesize ion transporters. The levels of acidic amino acid residues, such as aspartic acid and glutamic acid, increased. Furthermore, osmotic stress affected the translation, transcription, transport, and energy of *A. sclerotiales* and increased the proportion of alanine, glycine, and proline [9].

Moreover, fungi have adopted some strategies for coping with high salt stress. Intracellular ion homeostasis is essential for fungal survival and growth, and toxic Na^+^ excretion is a crucial strategy for fungi to cope with a high-salt environment. To maintain the osmotic pressure balance, the excess Na^+^ in the cell is discharged to the external through the Na^+^/H^+^ pump transmembrane protein, whereas the extracellular K^+^ is pumped into the cell by the K^+^ unidirectional transfer system [10,11]. However, ion transport and maintenance of osmotic balance is an energy-consuming process. Therefore, a sufficient energy supply is crucial for fungi. An increase in ATP production mediated through the control of aerobic respiration plays a central role in osmotic adaptation [12,13]. Fungi undergo different morphological changes to survive under a high-salt environment, such as cell wall thickening, decreased chitin content, increased β-glucan content, or β-glucan rearrangement in cell wall grids [14,15,16]. Moreover, *A. cristatus* [17,18], *A. montevidensis* [19], and *A. sydowii* [6] have adopted asexual reproduction strategies to adapt to high-salt environments. Among them, in a 5.13 M NaCl environment, the levels of antioxidant enzymes and lipid peroxidation biomarkers increased in *A. sydowii*, and the genes related to the cellular antioxidant defense system were also increased. This means that *A. sydowii* responded to hypertonic pressure. The oxidative stress system and antioxidant defense system were activated simultaneously [6].

*A. cristatus* is a crucial edible fungus [20,21,22,23] used for tea or substitute tea fermentation. During actual industrial fermentation, the environmental osmotic pressure greatly changes, thereby affecting the growth and material metabolism of the fungal strain as well as the quality and safety of fermented tea or substitute tea. However, the coping strategies and mechanisms of *A. cristatus* during this osmotic pressure change remain unclear. This paper reports the change in the rule of substance metabolism and metabolite synthesis of *A. cristatus* under different osmotic pressure environments created using NaCl.

## 2. Results and Analysis

### 2.1. Differences Were Observed in Pigment Substances Synthesized by A. cristatus under Different Osmotic Pressures

**Figure 1:**
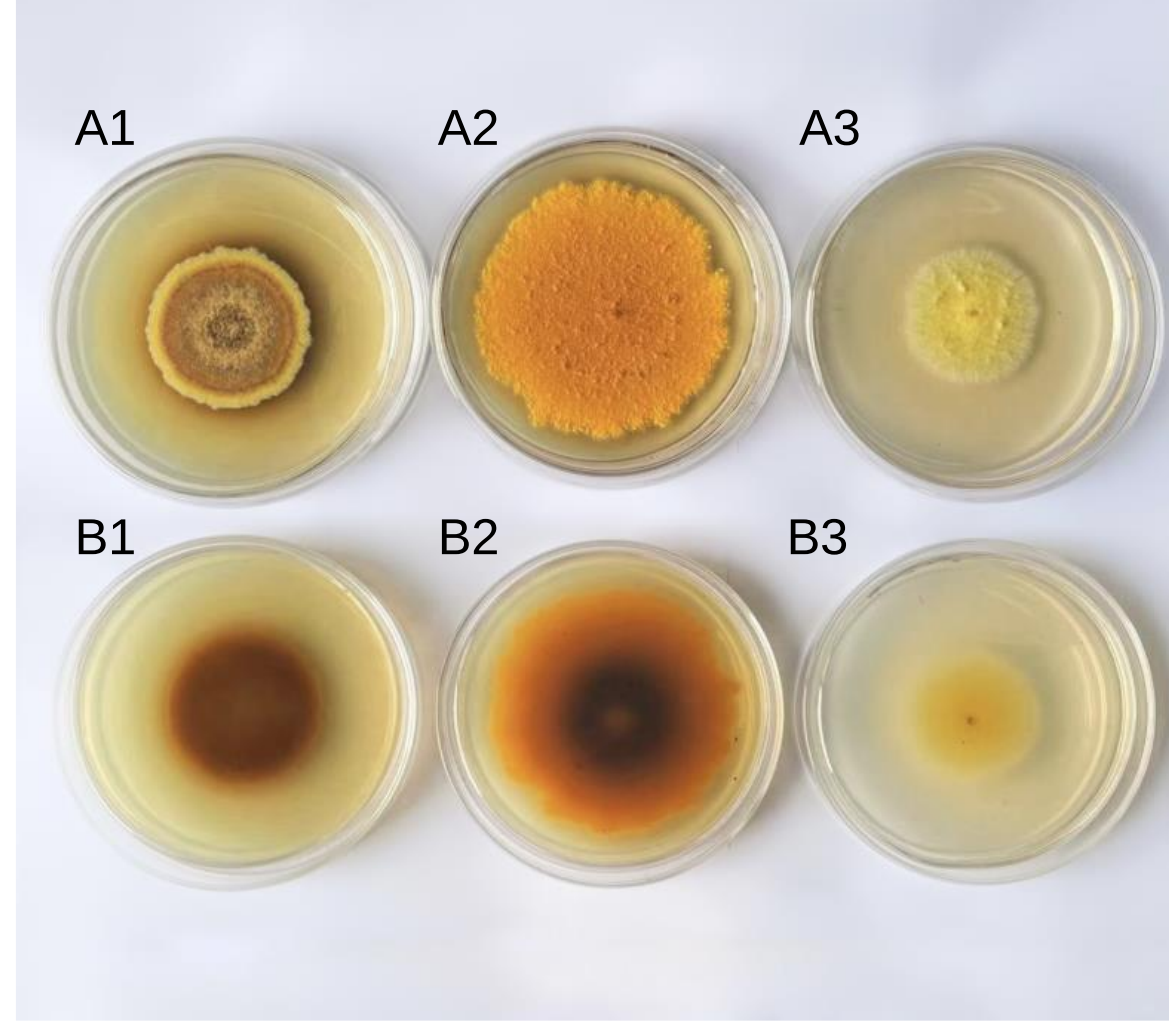
The colony morphology of *A. cristatus* growing on a medium containing different NaCl concentrations for 15 days. (**A1**–**A3**): The colony front of *A. cristatus* cultured for 15 days; (**B1**–**B3**): The opposite side of the colony of *A. cristatus* cultured for 15 days. (**A1**,**B1**) medium without NaCl colony; (**A2**,**B2**) medium containing 8% NaCl colony; and (**A3**,**B3**) medium containing 18% NaCl colony.

The *A. cristatus* strain was cultured at 25 °C for 15 days. The front of the colony grown on the medium containing no NaCl formed concentric rings of different colors, that is, bright yellow, khaki, light brown, dark yellow, and brown from the outside to the center, respectively. The front of the colony grown on the medium with 8% and 18% NaCl was orange and goose yellow, respectively, and no concentric rings of different colors were noted (Figure 1(A1–A3)). The color of the back of the *A. cristatus* colony on the medium containing 8% NaCl was orange in the outer ring and dark brown in the middle (Figure 1(B2)), and on the medium containing no NaCl and 18% NaCl was dark brown and goose yellow, respectively (Figure 1(B1,B3)). This result suggests that the fungal strain grows in media containing different NaCl concentrations, and the types or contents of pigments synthesized by the strain are different. Pigments are mostly secondary metabolites, although some are primary metabolites. Therefore, different NaCl concentrations are speculated to affect the material metabolic pathway and metabolic synthesis of *A. cristatus*.

### 2.2. Significant Differences Were Noted in the Mycelial Composition of A. cristatus Grown under Different Osmotic Pressures

**Figure 2:**
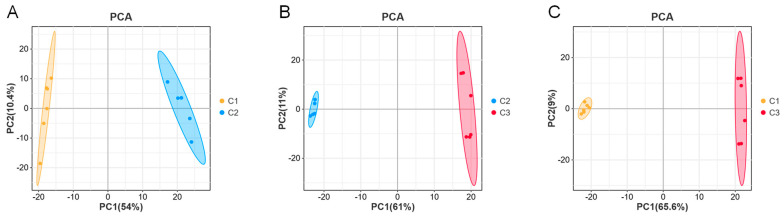
Principal component analysis (PCA). (**A**): PCA score plot (C1-C2); (**B**): PCA score plot (C2-C3); (**C**): PCA score plot (C1-C3). C1, C2, and C3 are mycelial samples of the strain cultured at 0%, 8%, and 18% NaCl, respectively.

The effect of NaCl concentration on the metabolic synthesis of *A. cristatus* was investigated through LC-MS-based non-targeted metabolomics analysis. Unsupervised PCA revealed that the six replicates of *A. cristatus* mycelial samples C1, C2, and C3 grown on media without NaCl and with 8% and 18% NaCl were clustered together and could be significantly separated. The differences between C1-C2, C2-C3, and C1-C3 were explained with probabilities of 64.4%, 72%, and 74.6%, respectively (Figure 2A–C). This suggests that the fungal strain grows on media with and without NaCl, and the synthesis of metabolites is different. The addition of different NaCl amounts also affects *A. cristatus* metabolism.

**Figure 3:**
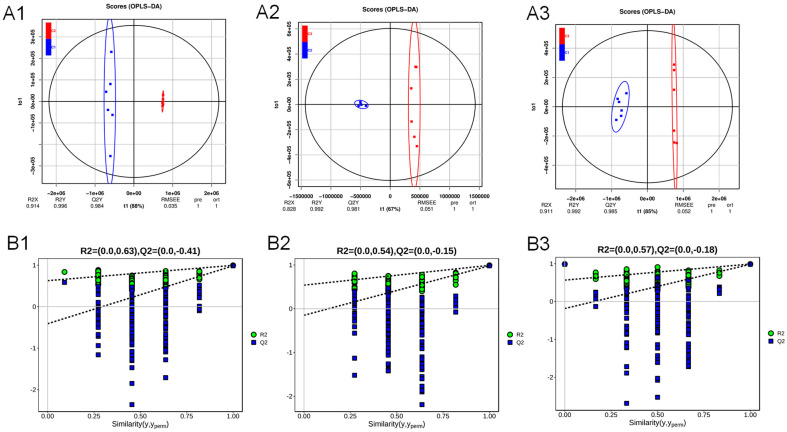
Orthogonal partial least squares discriminant analysis (OPLS-DA). C1-C2 (**A1**), C2-C3 (**A2**), and C1-C3 (**A3**) OPLS-DA model score scatter plot; the OPLS-DA model replacement test diagram of C1-C2 (**B1**), C2-C3 (**B2**), and C1-C3 (**B3**). C1, C2, and C3 are mycelial samples of the strain cultured at 0%, 8%, and 18% NaCl, respectively.

The supervised OPLS-DA analysis also demonstrated that the six replicates of mycelial samples C1, C2, and C3 achieved by growing *A. cristatus* on the medium without NaCl and with 8% and 18% NaCl were clustered together, distributed on the left and right sides, exhibiting a significant separation (Figure 3(A1–A3)). After the permutation test (Figure 3(B1–B3)), the intercepts of the Q2 regression line on the Y axis were all <0. This proves that the model is meaningful and can be explained in 88%, 67%, and 85% probabilities, respectively. The PCA and OPLS-DA analysis results suggested that the NaCl-induced change in the osmotic pressure of the medium significantly affected the material metabolism of *A. cristatus*. The mycelium of the fungal strain had different material composition and distribution patterns under different osmotic pressure conditions.

### 2.3. Different Substances of A. cristatus Mycelia under Different Osmotic Pressures

**Figure 4:**
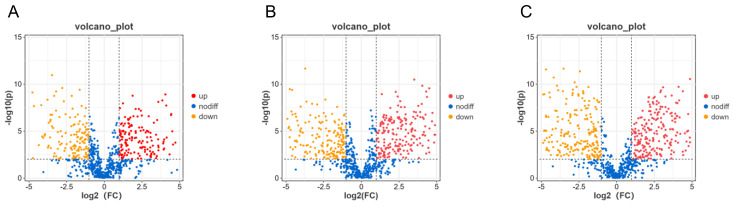
The analyses of differential substances for C1-C2, C2-C3, and C1-C3 using criteria set at the fold change (FC) > 2 or <0.5, and *p* < 0.05. (**A**–**C**): Volcano diagrams of differential substances between C1-C2 (**A**), C2-C3 (**B**), and C1-C3 (**C**). C1, C2, and C3 are mycelial samples of the fungal strain cultured at 0%, 8%, and 18% NaCl, respectively.

The 780 identified substances were screened based on the criteria of the fold change (FC) > 2 or <0.5 and *p* < 0.05. C1-C2 (Figure 4A), C2-C3 (Figure 4B), and C1-C3 (Figure 4C) produced 365, 454, and 515 differential substances, respectively. Among them, 365 identifiable substances in C1-C2 changed significantly, of which 193 and 172 substances were increased and reduced, respectively, as the NaCl concentration increased. Of the 454 differential substances in C2-C3, 229 and 225 substances were increased and reduced, respectively. From the 515 differential substances in C1-C3, 258 and 257 substances were increased and reduced, respectively, as the NaCl concentration increased. Moreover, differential substances mainly involved eight categories, namely flavonoids and flavonoids, organic oxygen compounds, amino acids and their derivatives, nucleosides, vitamins, toxins, hormones, and alkaloids.

**Figure 5:**
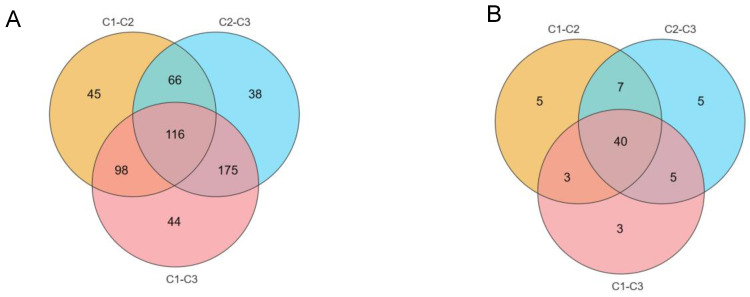
A Venn diagram of different substances and different metabolic pathways in C1-C2, C2-C3, and C1-C3. (**A**): Significantly different substances; (**B**): Significantly different metabolic pathways. C1, C2, and C3 are mycelial samples of the strain cultured at 0%, 8%, and 18% NaCl, respectively.

In total, 780 metabolites were identified from *A. cristatus* mycelial samples obtained from media with and without NaCl (Table S1). OPLS-DA and volcano plot analysis (set criteria: FC > 2 or 0.5, and *p* < 0.05), combined with the variable importance in projection (VIP) > 1, was used for the further screening of differential constituents. A total of 582 differential substances were analyzed. Among them, C1-C2, C2-C3, and C1-C3 exhibited 325 (Table S2), 395 (Table S3), and 433 (Table S4) differential substances, respectively. Additionally, C1-C2, C2-C3, and C1-C3 presented 116 common differential substances and had 45, 38, and 44 unique differential substances, respectively (Figure 5A). Compared with C1-C2 and C2-C3, C1-C3 had unique differences in substances, with the number of substances significantly increased and reduced being also very different. Therefore, the material metabolism of *A. cristatus* was believed to change significantly during the gradual increase in the medium NaCl concentration from 0% to 8% to 18%, and more than only one way of change existed.

#### 2.3.1. Flavonoids and Isoflavonoids

C1-C2 had 18 flavonoids as differential substances. Compared with C1, C2 had seven increased substances and eleven reduced substances. Among them, hispidulin, orientin, epicatechin, and formononetin were reduced by 461.83, 11.804, 11.02, and 10.111 times, respectively.

C2-C3 had nineteen flavonoids as differential substances. Compared with C2, C3 had twelve increased and seven reduced substances. Among them, the content of formononetin and cosmosiin was increased by 23.099 and 22.098 times, respectively. The contents of luteolin, sakuranetin, homoeriodictyol, and hesperetin were reduced by 129.19, 59.436, 19.875, and 16.568 times, respectively.

C1-C3 had 28 flavonoids as differential substances. Compared with C1, the contents of 16 substances were increased, whereas those of 12 substances were reduced. Among them, the content of cosmosiin was increased by 123.28 times; however, the contents of hispidulin, sakuranetin, luteolin, acacetin, homoeriodictyol, and hesperetin were reduced by 2576.9, 111.24, 102.26, 43.531, 38.465, and 27.7 times, respectively.

#### 2.3.2. Organic Oxygen Compounds

C1-C2 included 22 organic oxygen compounds. Compared with C1, fourteen substances were increased, and eight were reduced in C2. Among them, the contents of D-arabitol and fructose-1,6-diphosphate were increased by 95.562 and 53.666 times, whereas the contents of l-erythrulose and erythritol were reduced by 13.482 and 3.0564 times, respectively.

C2-C3 included 27 organic oxygen compounds. Compared with C2, eight substances were increased, and nineteen were reduced in C3. Among them, the contents of l-ribulose and 3-hydroxybenzyl alcohol glucoside were increased by 15.3 and 6.383 times, whereas that of trehalose, D-mannose, fructose-1,6-diphosphate, erythritol, and D-arabitol were reduced by 24.628, 7.477, 3.459, 3.081, and 2.818 times, respectively.

C1-C3 included 35 organic oxygen compounds. Compared with C1, 11 substances were increased, and 24 were reduced in C3. Among them, the contents of D-arabitol and fructose-1,6-diphosphate were increased by 33.916 and 15.516 times, respectively, whereas the contents of trehalose, l-erythrulose, erythritol, and D-mannose were reduced by 40.187, 15.402, 9.417, and 6.399 times respectively.

#### 2.3.3. Amino Acids and Their Derivatives

C1-C2 had 43 amino acids and their derivatives. Compared with C1, C2 had 23 increased and 20 reduced substances. Among them, the contents of *N*-formyl-l-methionine, glycine-leucine, β-hydroxyarginine, glutathione, aspartame, and L-asparagine were increased by 123.12, 26.637, 20.353, 17.512, 15.209, and 9.909 times, respectively. The contents of l-prolinamide, l-histidine, and glycitein were reduced by 40.929, 11.659, and 9.035 times, respectively.

C2-C3 had 52 amino acids and their derivatives. Compared with C2, 33 substances were increased, and 19 substances decreased in C3. The contents of l-methionine, glutaric acid, γ-glutamylcysteine, *N*,*N*-dimethylhistidine, and l-prolinamide were increased by 101.84, 84.215, 41.634, 36.515, and 5.505 times, respectively. In contrast, the contents of β-hydroxyarginine, *N*(6)-[(indol-3-yl)acetyl]-l-lysine, *S*-adenosylmethionine, l-asparagine, pyroglutamic acid, glycyl-leucine, and glutathione were reduced by 378.9, 277.74, 64.964, 13.153, 12.7, 7.903, and 5.571 times, respectively.

C1-C3 had 48 amino acids and their derivatives. Compared with C1, C3 had 30 increased and 18 reduced substances. The contents of *N*-formyl-l-methionine, l-methionine, *N*,*N*-dimethylhistidine, γ-glutamylcysteine, and glutathione were increased by 393.1, 43.299, 33.913, 14.163, and 3.143 times, respectively. In contrast, the contents of *N*(6)-[(indol-3-yl)acetyl]-l-lysine, pyroglutamic acid, l-histidine, and *S*-adenosylmethionine were reduced by 192.69, 24.528, 22.257, and 21.222 times, respectively.

#### 2.3.4. Nucleosides

C1-C2 had 20 nucleoside differential substances. Compared with C1, C2 had fifteen increased and five reduced substances. Among them, uridine, 2′-deoxyadenosine, nicotinamide riboside, uracil, UMP, and 5′-methylthioadenosine were increased by 61.599, 23.13, 16.828, 15.945, 15.498, and 12.069 times, respectively, whereas guanosine was reduced by 15.432 times.

C2-C3 had 27 nucleoside differential substances. Compared with C2, C3 had 15 increased and 12 reduced substances. ADP, uridine 5′-diphosphate (UDP), and nicotinamide ribotide were increased by 71.287, 19.086, and 17.504 fold, respectively. In contrast, cytosine, 5,6-dihydro-5-fluorouracil, uridine diphosphate glucose, and uracil were reduced by 257.59, 29.62, 20.543, and 17.059 times, respectively.

C1-C3 had a total of 32 nucleoside differential substances. C3 had 21 increased and 11 reduced substances compared with C1. Uridine, UDP, 2′-deoxyadenosine, ADP, GMP, and UMP were increased by 126.02, 98.677, 80.378, 46.9, 29.259, and 15.226 fold, respectively. Cytosine and 5,6-dihydro-5-fluorouracil were reduced by 177.91 and 176.48 times, respectively.

#### 2.3.5. Vitamins

C1-C2 had five different vitamins. Compared with C1, C2 had three increased and two reduced substances. Pantothenol, pantothenic acid, and thiamine were increased by 77.944, 8.619, and 2.088 times, respectively. Rutin and niacinamide were reduced by 26.413 and 2.898 times, respectively.

C2-C3 had four different vitamins. Compared with C2, C3 had two increased and two reduced substances. Dehydroascorbate and nicotinic acid were increased by 29.946 and 12.022 times, respectively. Thiamine and niacinamide were reduced by 52.61 and 5.101 times, respectively.

C1-C3 had seven vitamin different vitamins. Compared with C1, C3 had four increased and three reduced substances. Pantothenol, dehydroascorbate, pantothenic acid, and nicotinic acid were increased by 64.077, 29.591, 12.414, and 5.394 times, respectively. Rutin, thiamine, and niacinamide were reduced by 56.843, 25.2, and 14.785 times, respectively.

#### 2.3.6. Toxins

C1-C2 had two toxin differential substances, and the alternariol and aflatoxin G2 contents in C2 were 12.214 and 2.059 times higher than in C1.

C2-C3 had three toxin differential substances, and the contents of aflatoxin G1, T2 toxin, and alternariol were increased by 56.178, 16.927, and 10.371 times in C3 compared with C2.

C1-C3 had four toxin differential substances. Compared with C1, C3 had three increased substances and one reduced substance. The contents of alternariol, aflatoxin G1, and T2 toxin were increased by 126.68, 88.779, and 7.289 times, respectively. In contrast, citrinin was reduced by 145.48 times.

#### 2.3.7. Hormone Classes

C1-C2 had 21 hormone differential substances. Compared with C1, C2 had 11 increased and 10 reduced substances. Among them, 4′-O-methylnorbelladine, quinestrol, and gibberellin A124 were increased by 66.989, 23.938, and 5.439 times, respectively. Metaproterenol, prostaglandin C2, prostaglandin A2, and gibberellin A3 were reduced by 336.22, 69.985, 3.066, and 2.388 times, respectively.

C2-C3 had 27 hormone differential substances. Compared with C2, C3 had nine increased and sixteen reduced substances. Luteoforol, prostaglandin C2, and 11-dehydrocorticosterone were increased by 30.277, 26.131, and 22.061 times, respectively. Gibberellin A3 and A124 and prostaglandin A2 were reduced by 653.08, 60.727, and 20.647 times, respectively.

C1-C3 had 34 hormone differential substances. Compared with C1, C3 had 11 increased and 23 reduced substances. Prostaglandin G2, matricarin, and androsterone were increased by 21.642, 21.273, and 21.27 times, respectively. Gibberellin A3, prostaglandin A2, and gibberellin A124 were reduced under hypertonic conditions by 1559.7, 63.299, and 11.166 times, respectively.

#### 2.3.8. Alkaloids

C1-C2 had 20 alkaloid differential substances. Compared with C1, C2 had eleven increased and nine reduced substances. The contents of fluvastatin and tryptophanol increased by 21.802 and 10.983 times, respectively. The content of isopyridoxal was reduced by 47.598 times.

C2-C3 had 19 alkaloid differential substances. Compared with C2, C3 had eleven increased and eight reduced substances. The contents of albendazole, fluvastatin, and isopyridoxal were increased by 15.223, 12.141, and 2.923 times, respectively. The contents of tryptophanol, amodiaquine, and indoleacetaldehyde were reduced by 713.88, 107.19, and 37.011 times, respectively.

C1-C3 had 25 alkaloid differential substances. Compared with C1, C3 had 13 increased and 12 reduced substances. Fluvastatin and albendazole were up-regulated by 264.71 and 144.31 times, respectively. In contrast, amodiaquine, tryptophanol, isopyridoxal, and indoleacetaldehyde were reduced by 105.42, 64.998, 16.282, and 11.08 times, respectively.

### 2.4. Metabolic Pathway Analysis

Among the C1-C2, C2-C3, and C1-C3 samples, 133 metabolic pathways were involved (Table S5). A total of 66 differential metabolic pathways were screened, mainly involving seven categories: amino acid metabolism, vitamin metabolism, nucleotide metabolism, lipid metabolism, carbon metabolism, energy metabolism, and secondary metabolite metabolic pathways.

Among them, C1-C2, C2-C3, and C1-C3 involved 55, 57, and 51 differential metabolic pathways, respectively. They had 5, 5, and 3 unique differential metabolic pathways and 40 differential metabolic pathways (Figure 5B). The metabolic pathways mainly included 16 amino acid metabolic pathways such as alanine, histidine, aspartic acid, and glutamic acid metabolism, four nucleotide metabolic pathways such as purine and pyrimidine, and nine secondary metabolite pathways such as zeatin and steroid hormones. Furthermore, porphyrin, carbon, and fatty acid metabolic pathways changed significantly as the NaCl concentration in the medium increased.

### 2.5. Antioxidant Activity and Correlation Analysis of A. cristatus under Different Osmotic Pressures

#### 2.5.1. Significant Differences Were Noted in the Antioxidant Activity of *A. cristatus* Mycelia under Different Osmotic Pressures

**Figure 6:**
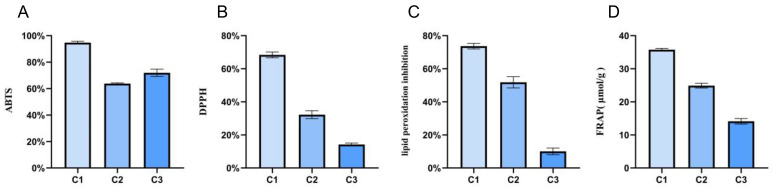
Evaluation of antioxidant activity of *A. cristatus* under different osmotic pressures. (**A**): ABTS free-radical scavenging rate; (**B**): DPPH free-radical scavenging rate; (**C**): lipid peroxidation inhibition rate; (**D**): Ferric ion-reducing antioxidant power. C1, C2, and C3 are mycelial samples of the strain cultured at 0%, 8%, and 18% NaCl, respectively.

The aforementioned results revealed that the metabolism of *A. cristatus* was regulated by osmotic pressure. The metabolites included primary and secondary metabolites. Some metabolites exhibit antioxidant activity. Therefore, this study used an in vitro antioxidant model to explore the antioxidant activity of *A. cristatus* under different osmotic pressures. The results unveiled (Figure 6) that the ABTS and DPPH free-radical scavenging, lipid peroxidation inhibition, and ferric ion-reducing antioxidant power of mycelium C1 grown on the medium containing no NaCl were significantly higher than those of mycelia C2 and C3 grown on the medium containing 8% and 18% NaCl (*p* < 0.0001). Among them, the ABTS and DPPH free-radical scavenging, lipid peroxidation inhibition, and ferric ion-reducing antioxidant power of mycelium C1 were as high as 96%, 68%, 75%, and 36 μmol/g, respectively. The ABTS free-radical scavenging of mycelium C3 was higher than that of mycelium C2, and the other three antioxidant capacities of mycelium C3 were significantly lower than those of mycelium C2 (*p* < 0.0001). The DPPH free-radical scavenging, lipid peroxidation inhibition, and ferric ion-reducing antioxidant power of mycelium C3 were only 15%, 10%, and 14 μmol/g.

#### 2.5.2. Correlation Analysis

**Figure 7:**
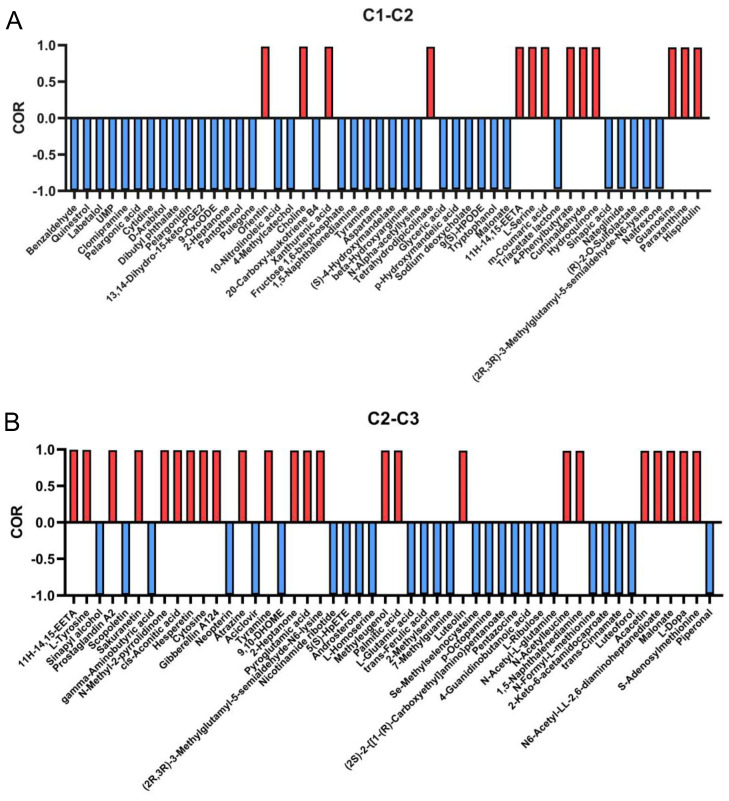
Correlation analysis between the differential substances and the antioxidant activities. Correlation analysis of important substances in C1-C2 (**A**), C2-C3 (**B**), and lipid peroxidation inhibition rate. C1, C2, and C3 are mycelial samples of the fungal strain cultured at 0%, 8%, and 18% NaCl, respectively.

Several antioxidant activities of *A. cristatus* mycelia changed with a change in osmotic pressure in the growth environment. Here, we further explored the material basis that leads to changes in the antioxidant activity of mycelia. The aforementioned results showed that the DPPH free-radical scavenging, lipid peroxidation inhibition, and ferric ion-reducing antioxidant power of *A. cristatus* mycelia exhibited a consistent change rule with a change in the environmental osmotic pressure from low to high. In this paper, data on lipid peroxidation inhibition were used to present the results of correlation analysis. Therefore, the correlation between the differential substances identified in C1-C2 and C2-C3 and the lipid peroxidation inhibition was analyzed (Table S6), and 50 substances with the greatest correlation were screened out (Figure 7). Among them, 13 substances were positively correlated with an improvement in the antilipid peroxidation ability of the C1 mycelium, such as orientin, choline, xanthurenic acid, l-serine, guanosine, paraxanthine, and hispidulin. These 13 substances are believed to be crucial contributors to the significant improvement in the antilipid peroxidation ability of the C1 mycelium. That is, when the environmental osmotic pressure increased to 8% NaCl, the ability of *A. cristatus* to metabolize and synthesize these substances was hindered. In addition, other substances also contribute to antilipid peroxidation. For example, the ability of *A. cristatus* to metabolize and synthesize l-tyrosine, prostaglandin A2, sakuranetin, cis-aconitic acid, cytosine, gibberellin A124, pyroglutamic acid, luteolin, *N*-acetylleucine, and *S*-adenosylmethionine was also significantly reduced when the environmental osmotic pressure continued to increase to 18%NaCl. These results suggest that the regulation of environmental osmotic pressure by *A. cristatus* on the synthesis of different antilipid peroxidation active substances is different.

## 3. Discussion

### 3.1. From Low to High Osmotic Pressure, Various Substances Changed Significantly in A. cristatus, and the Change Intensity Was Large and Involved Seven Main Change Modes

Under high salt stress, cells undergo physiological reactions such as osmotic pressure imbalance, metabolic disorders, oxidative stress, and ion toxicity, and fungal cells are no exception. Under high salt stress, a series of changes in metabolic pathways occur in fungal cells that are essential for survival [4,7]. In our study, the material and metabolic pathways of *A. cristatus* changed significantly when the NaCl concentration changed from low to high. The changed substances mainly involved amino acids and their derivatives, flavonoids, organic oxygen compounds, nucleosides, vitamins, toxins, hormones, and alkaloids. The number of substances that significantly changed were 143, 65, 84, 79, 16, 9, 82, and 64, and the variation ranges were 393.1–2.039, 2576.9–2.033, 95.562–2.039, 257.59–2.011, 77.944–2.088, 145.48–2.059, 1559.7–2.33, and 713.88–2.060, respectively. Among them, the maximum changes in flavonoids, isoflavonoids, and hormones, such as hispidulin and gibberellin A3, could be thousands of times. Hispidulin and gibberellin A3 were reduced by 2576.9 and 1559.7 times, respectively, without NaCl compared with 18% NaCl.

Moreover, as the NaCl concentration increased, metabolic changes in *A. cristatus* differed. According to data on differential substance changes, seven main types of change patterns existed. The first category had 116 substances that were significantly different in C1-C2, C2-C3, and C1-C3, such as d-arabitol, fructose-1,6-diphosphate, erythritol, glutathione, thiamine, nicotinamide, alternariol, gibberellin A3, prostaglandin A2, fluvastatin, and tryptophan. These substances exhibited continuous significant upregulation or continuous significant downregulation with NaCl-induced increase in osmotic pressure. The second category had 45 substances that changed significantly only in C1-C2 when the NaCl concentration increased from 0% to 8%, but not in C2-C3 and C1-C3, such as epicatechin, 6-phosphate glucose, oxalic acid, l-malic acid, and 3-*O*-methylquercetin. These substances were significantly increased or reduced when the NaCl concentration increased from 0% to 8%. The substances were also reduced or increased when the NaCl concentration increased from 8% to 18%, but not significantly. The third category included 38 substances that only changed significantly in C2-C3, such as leucine, d-glycerol-d-galactose-peptide alcohol, (*S*)-abscisic acid, deoxyinosine, valeric acid, 2′-hydroxygenistein, and d-phenylalanine. These substances changed when the NaCl concentration increased from 0% to 8%. The substances were increased or reduced, but the changes were not significant. However, when the NaCl concentration increased from 8% to 18%, these substances were reduced or increased significantly. In the fourth category, 44 substances were unique to C1-C3, such as l-isoleucine, d-galactose, guanosine 3′-phosphate, luteolin, and naringin. When the NaCl concentration increased from 0% to 8% and then increased to 18%, the synthesis of these substances increased or decreased, not significantly, but the accumulation of two consecutive upregulation or downregulation was significant. The fifth category includes 66 substances. These substances are common differential metabolites between C1-C2 and C2-C3. They were significantly increased or reduced as the NaCl concentration increased from 0% to 8%. When the NaCl concentration increased from 8% to 18%, these substances were significantly reduced or increased to an extent similar to that when the NaCl concentration increased from 0% to 8%. These substances included 2-heptanone, l-serine, citric acid, genistein, l-aspartic acid, d-guanine, glycine, leucine, and ornithine. The sixth category included 175 substances, which were the common differential metabolites of C1-C3 and C2-C3. These substances exhibited no significant change when the NaCl concentration increased from 0% to 8% but changed significantly with an increase from 8% to 18%. The substances included aflatoxin G1, luteolin, T2 toxin, glutamic acid, nicotinic acid, estradiol, *S*-methyl-l-methionine, and deoxyguanosine. The seventh category included 98 substances, which were common differential metabolites between C1-C2 and C1-C3 samples. These substances changed significantly with an increase in the NaCl concentration from 0% to 8%. No significant changes in these substances were observed when the NaCl concentration increased from 8% to 18%. The seventh category of substances included rutin, l-histidine, nicotinamide ribose, l-arginine, l-erythrulose, neochlorogenic acid, glycine, and thymine.

The aforementioned discussion shows that when the NaCl-induced osmotic pressure of the growth environment changes from low to medium to high, many types of substances undergo significant metabolic changes, and the change range is large. Seven main modes of change exist. The changed metabolites are involved in primary and secondary metabolism. A similar report has not been observed in other fungal studies.

### 3.2. Arabitol and Fructose-1,6-diphosphate Are the Main Osmotic Regulators of A. cristatus in Response to a High-Salt Environment

Under high salt stress, cells tend to accumulate compatible solutes as osmotic regulators (1–5). In a high-salt environment, fungi maintain osmotic balance by accumulating high-energy metabolites (15). In our study, *A. cristatus* accumulated trehalose, erythritol, and l-erythrulose when grown without NaCl. This result is consistent with those reported for *A. sydowii*. However, when grown in the 8% and 18% NaCl environment, unlike *A. sydowii*, which accumulated glycerol, *A. cristatus* significantly accumulated arabitol and fructose-1,6-diphosphate. Fructose-1,6-diphosphate is mainly enriched in three KEGG pathways: carbon metabolism, carbon fixation in photosynthetic organisms, and methane metabolism, and the metabolic pathway flux was increased. Moreover, *A. montevidensis* mainly accumulates soluble sugars and saturated fatty acids (7) in a high-salt environment. This shows that in a high-salt environment, *A. cristatus* possesses a set of osmotic regulation strategies different from those of *A. sydowii* and *A. montevidensis*. Arabinitol and fructose-1,6-diphosphate are osmotic regulators for *A. cristatus* to cope with high osmotic pressure.

### 3.3. A. cristatus Reduced the Growth Rate in a High-Salt Environment, Accumulated Survival Competitive Substances, and Initiated Oxidative Stress and Antioxidant Defense System

In our study, *A. cristatus* greatly limited the synthesis of gibberellin A3 and A124, which are related to vegetative growth, in a high-salt environment. Gibberellin A3 was especially reduced by 1559 times at 18% NaCl compared with no NaCl. In addition, the content of prostaglandin A2 [24], which can inhibit cell proliferation, was reduced by 63 times under high salt conditions. The decrease in gibberellin A3 and A124 and prostaglandin A2 synthesis may mainly cause the slow vegetative growth of *A. cristatus* in a high-salt environment. This means high salt can regulate hormone metabolism related to *A. cristatus* growth.

Fungi not only control the growth rate under a high-salt environment but also activate the oxidative stress system and antioxidant defense system [25,26,27] to cope with the lethal effects of high salt-induced high osmotic pressure on cells, such as *A. sydowii* [6]. In our study, at 18% NaCl, the content of UDP in *A. cristatus* was 98 times higher than that in the absence of NaCl. UDP is an endogenous signal molecule produced by damaged cells, which suggests that *A. cristatus* cells were damaged when exposed to a high-salt environment. At the same time, large amounts of antioxidant substances such as glutathione, dehydroascorbate, and cosmosiin accumulated at 18% NaCl. These substances were 3, 29, and 123 times higher than those in the absence of NaCl, respectively. UDP was mainly enriched in three significantly different metabolic pathways: zeatin biosynthesis, pyrimidine metabolism, and nucleotide metabolism. Glutathione is a significantly different metabolite in glutathione metabolism and cysteine and methionine metabolism. However, some other antioxidant active substances, such as hispidulin [28,29], sakuranetin [30], hesperetin [31], rutin [32], and homoeriodictyol [33], were significantly reduced, especially in crude ragweed. The content of ragweed was reduced by 2576 times, suggesting that *A. cristatus* also has a set of mechanisms for the oxidative stress system and antioxidant defense in response to high-salt osmotic stress. However, the antioxidant active substances for repairing oxidative stress damage were mainly glutathione [34], dehydroascorbate [35], and cosmosiin.

Fungi enhance energy metabolism in response to hyperosmotic stress [3,12,13,36]. With 18% NaCl, pantothenol and pantothenic acid, which are related to energy metabolism, were increased by 64 and 12 times, respectively, compared with without NaCl. Moreover, with NaCl, alternariol, aflatoxin G1, aflatoxin T2, and fluvastatin [37,38] were increased by 126, 88, 7, and 264 times, respectively, compared with without NaCl. These substances exhibit a wide range of antibacterial activity, indicating that *A. cristatus* accumulates substances related to survival competition in a high-salt environment to maintain survival space and nutritional needs.

## 4. Materials and Methods

### 4.1. Strains and Reagents

#### 4.1.1. Strains

*A. cristatus* CGMCC 3.17718 was purchased from the China General Microbiological Culture Collection and Management Center (Beijing, China).

#### 4.1.2. Medium

Modified Czapek’s medium containing (NH4)_2_SO_4_ 3 g, K_2_HPO_4_ 1 g, MgSO_4_ 0.5 g, KCl 0.5 g, FeSO_4_ 0.01 g, sucrose 30 g, agar 16 g, and H_2_O 1 L and of neutral pH was sterilized at 121 °C for 20 min.

#### 4.1.3. Main Reagents

The reagents used in the study were NaCl (≥99.5%, Aladdin, Los Angeles, SC, USA), 2,2′-azino-bis(3-ethylbenzothiazoline-6-sulfonate (ABTS) (≥98.0%, Aladdin, Los Angeles, SC, USA), 1,1-diphenyl-2-picrylhydrazyl (DPPH) (≥97.0%, Tokyo Seiko, Shiki Park, Minato Ward, Tokyo, Japan), ethanol (≥99.8%, Aladdin, Los Angeles, SC, USA), trichloroacetic acid (TCA) (≥99.0%, Aladdin, Los Angeles, SC, USA), thiobarbituric acid (TBA) (≥98.0%, Amresco, Solon Industrial Parkway Solon, Solon, OH, USA), FeSO_4_·7H_2_O (≥99.0%, Amresco, Solon Industrial Parkway Solon, OH, USA), Methanol (≥99.0%, ThermoFisher, Waltham, MA, USA), acetonitrile (≥99.9%, ThermoFisher), 2-amino-3-(2-chloro-phenyl)-propionic acid (98.5%, Aladdin, Los Angeles, SC, USA), formic acid (LC–MS Grade, TCI, Tokyo, Japan), ammonium formate (≥99.9%, Sigma, St. Louis, MO, USA), and H_2_O (Millipore, Burlington, MA, USA). All reagents were analytically pure.

### 4.2. Main Instruments and Equipment

Vertical pressure steam sterilization pot (LDZX-50 KBS, Shanghai, China), mold incubator (TAISITE, MJX-series, Tianjin, China), high-speed freezing centrifuge (Beckman, Allegra x-30r, Brea, CA, USA), vortex mixer (Kylin-bell, BE-2600, Haimen, China), tissue grinder (Meibi, MB-96, Jiaxing, China), ultrasonic cleaner (Shumei, KQ-800DE, Kunshan, China), liquid chromatograph (Thermo, Vanquish, Waltham, MA, USA), mass spectrometer (Thermo, Orbitrap Exploris 120, Waltham, MA, USA), vacuum concentrator (Eppendorf, 5305, Hamburg, Germany) and digital display constant temperature water bath (MX15H135-A12E, Niles, IL, USA) were all used in this study.

### 4.3. Culture Method of A. cristatus on Different Osmotic Pressure Media

#### 4.3.1. Preparation Method of Different Osmotic Pressure Media

The osmotic pressure of the medium was adjusted by adding different NaCl concentrations. NaCl with a mass volume ratio (*m*/*v*) of 0%, 8%, and 18% was added to prepare modified Czapek’s medium with different NaCl concentrations. The prepared culture medium was sterilized at 121 °C for 20 min, and the pressure was ≤0.24 MPa.

#### 4.3.2. Culture of *A. cristatus* on Different Osmotic Pressure Media and Mycelium Preparation Method

*A. cristatus* strains were inoculated in the center of the plate containing different NaCl concentrations. Then, the strains were cultured in a constant temperature and humidity incubator at 25 °C and 65–70% RH for 15 days, and 6 replicates were established for each medium.

The mycelia were collected in a sterile environment, transferred to a sterilized Eppendorf tube, and refrigerated at −80 °C until used for subsequent metabolomics analysis.

### 4.4. Determination of Antioxidant Capacity

First, 0.1 g *A. cristatus* mycelia was accurately weighed, and 2 mL distilled water was added. Then, the mycelia were subjected to an ultrasonic cleaner (Shumei, KQ-800DE) for 30 min at 30 °C to ultrasonically extract the content and centrifuged for 10 min at 10,000 rpm and 4 °C. The supernatant was collected.

#### 4.4.1. Assessment of ABTS Free-Radical Scavenging

ABTS free-radical scavenging was assessed following the method of Xiao et al. [39].

#### 4.4.2. Assessment of DPPH Free-Radical Scavenging

DPPH free-radical scavenging was assessed following the method of Aniya et al. [40].

#### 4.4.3. Assessment of Lipid Peroxidation Inhibition

Lipid peroxidation inhibition was assessed following the method of Wu et al. [41].

#### 4.4.4. Assessment of Ferric Ion-Reducing Antioxidant Power

The ferric ion-reducing antioxidant power was assessed using the Total Antioxidant Capacity Assay Kit of Solarbio (Total Antioxidant Capacity(T-AOC), Solarbio, Beijing, China).

### 4.5. LC–MS Non-Targeted Metabolomics Detection Method

Reference https://www.ncbi.nlm.nih.gov/pmc/articles/PMC3772505/ (accessed on 20 March 2024), The reporting standards for metabolomics experiments include steps such as sample preparation, data preprocessing, quality control, and metabolite identification.

#### 4.5.1. Sample Preparation

First, 60 mg *A. cristatus* mycelia was accurately weighed and added to a 2 mL centrifuge tube. Then, 100 mg glass beads and 1000 µL of 50% methanol–water (stored at 4 °C) were added to the tube and vortexed for 30 s using a vortex mixer (Kylin-bell, BE-2600). The centrifuge tube containing the sample was placed in the 2 mL adapter matched with the instrument, immersed in liquid nitrogen for rapid freezing for 5 min, thawed at room temperature, and again placed in the 2 mL adapter. The adapter was installed into the tissue grinder (Meibi, MB-96), and the sample was ground at 55 Hz for 2 min. The mycelium was first repeatedly frozen, then thawed, and finally ground twice. The centrifuge tube was removed and centrifuged for 10 min at 12,000 rpm and 4 °C. All the supernatant was collected and transferred to a new 2 mL centrifuge tube. The supernatant was concentrated and dried using a vacuum concentrator (Eppendorf, 5305). Then, 300 µL of 2-amino-3-(2-chloro-phenyl)-propionic acid (4 ppm) solution prepared with 50% methanol–water (stored at 4 °C) was accurately added to redissolve the sample. The supernatant was filtered using a 0.22 μm membrane and transferred into the detection vial for LC–MS detection.

#### 4.5.2. Liquid Chromatography Conditions

The LC analysis was conducted on a Vanquish UHPLC System (Thermo Fisher Scientific, Waltham, MA, USA). Chromatography was performed using the ACQUITY UPLC^®^ HSS T3 column (150 × 2.1 mm, 1.8 µm) (Waters, Milford, MA, USA) maintained at 40 °C. The flow rate and injection volume were 0.25 mL/min and 2 μL, respectively. For the LC-ESI (+)-MS analysis, the mobile phases consisted of (C) 0.1% formic acid in acetonitrile (*v*/*v*) and (D) 0.1% formic acid in water (*v*/*v*). Separation was performed using the following gradient: 0–1 min, 2% C; 1–9 min, 2–50% C; 9–12 min, 50–98% C; 12–13.5 min, 98% C; 13.5–14 min, 98–2% C; 14–20 min, 2% C. The LC-ESI (−)-MS analysis was conducted using (A) acetonitrile and (B) ammonium formate (5 mM). Separation was performed using the following gradient: 0–1 min, 2% A; 1–9 min, 2–50% A; 9–12 min, 50–98% A; 12–13.5 min, 98% A; 13.5–14 min, 98–2% A; 14–17 min, 2% A.

#### 4.5.3. Mass Spectrum Conditions

Mass spectra of the metabolites were determined using Orbitrap Exploris 120 (Thermo Fisher Scientific, USA) with an ESI ion source. Simultaneous MS1 and MS/MS (Full MS-ddMS2 mode, data-dependent MS/MS) acquisition was performed. The parameters were as follows: sheath gas pressure, 30 arb; aux gas flow, 10 arb; spray voltage, 3.50 kV and −2.50 kV for ESI(+) and ESI(−), respectively; capillary temperature, 325 °C; MS1 range, *m*/*z* 100–1000; MS1 resolving power, 60,000 FWHM; number of data-dependent scans per cycle, 4; MS/MS resolving power, 15,000 FWHM; normalized collision energy, 30%; dynamic exclusion time, automatic.

#### 4.5.4. Data Preprocessing

Original LC–MS data were obtained using Proteowizard 3.0.9134 software and converted into the mzXML format. The XCMS program package of R was used for peak recognition, peak filtering, and peak alignment. The data matrix, including the plasma–nucleus ratio (*m*/*z*), retention time, and peak area (intensity), was obtained. Then, normalized preprocessing was performed, followed by filtering out characteristic peaks with NA exceeding 50% in quality control (QC) samples and substances with NA exceeding 80% in all samples. Subsequently, missing values were filled in based on individual substances and all samples (KNN filling). Finally, after QC normalization, the QC samples were set up (each sample was taken in equal quantities and mixed). The QC process retains substances in the QC samples with a coefficient of variation of <30% for the subsequent analysis.

#### 4.5.5. Metabolite Identification

First, the first-level qualitative analysis was conducted based on the mass-to-charge ratio and retention time. According to the results of the first-level analysis, the fragment information of metabolites in the sample was compared with that available on the database for the second-level qualitative analysis. In the second-level analysis, algorithms with different dimensions were established. Ultimately, all algorithms were comprehensively considered, including those for fragment matching, fragment relative response, ppm, and other parameters, to provide the final identification result. Generally, the molecular weight error is ppm <30, and the comprehensive score is >0.6.

### 4.6. Statistics and Multivariate Analysis

Multivariate statistical analyses, such as principal component analysis (PCA) and orthogonal partial least squares discriminant analysis (OPLS-DA), were performed using the microbiome cloud platform (https://www.bioincloud.tech and https://www.omicshare.com/ accessed on 1 June 2023), to monitor metabolic changes during *A. cristatus* growth. PCA was performed to fit overall data, detect internal changes in these data matrices, and distinguish the differences between *A. cristatus* samples. OPLS-DA was conducted to categorize samples with only Y variables. The omicshare cloud platform was used to analyze the volcano map and Venn diagram. By calculating the fold change (FC) of metabolites, we could determine whether the metabolites have changed between the groups, the extent of change and whether the difference is significant. Combined with the *p*-value, some crucial metabolites can be screened out. Combined with the difference in the weight contribution value (VIP) (The contribution of metabolites with VIP > 1 to the discriminant analysis was larger, and these metabolites were significantly different between the groups), substances contributing significantly to the differentiation of varying samples were screened. The topological analysis and enrichment analysis were combined to screen for key metabolic pathways. First, metabolites with *p* < 0.05 were screened through a *t*-test, and then, the metabolic pathways in which these metabolites appeared were identified. By calculating the Over Representation Analysis *p*-values of these metabolic pathways, we identified the significant enrichment pathways of differential metabolites. Finally, topological analysis was performed to calculate the magnitude of the effect of metabolites in the metabolic pathways (measured by impact). For the correlation analysis, the identified compounds were used as X variables, whereas the antioxidant activity evaluation results were used as Y variables. We also observed whether the correlation of metabolites changes between different groups.

## 5. Conclusions

*A. cristatus* adapts to changes in salt concentration by regulating metabolic pathways and their metabolite synthesis. Moreover, their material change patterns are diverse. The fungus implements unique strategies to tackle high salt stress, including down- and upregulation of synthesis of some substances, growth restriction, osmotic pressure balance, oxidative stress response, antioxidant defense, and survival competition.

*A. cristatus* mainly downregulates gibberellin A3 and A124 and prostaglandin A2 synthesis to limit growth. The osmotic balance is maintained by increasing the metabolic flux and accumulating a large amount of arabitol and fructose-1,6-diphosphate. Moreover, energy metabolism is enhanced by upregulating pantothenol and pantothenic acid synthesis, and the antioxidant defense system involving glutathione, dehydroascorbate, and cosmosiin is activated. To achieve competitive survival with other organisms, numerous substances with antibacterial activity are also synthesized, such as fluvastatin, aflatoxin, and alternariol.

The contents of both primary and secondary metabolites of *A. cristatus* were increased or reduced with a change in osmotic pressure; that is, environmental osmotic pressure can regulate the metabolism of *A. cristatus*. Therefore, osmotic pressure can be used as a major substance metabolism regulator in the actual *A. cristatus* fermentation process.

## Data Availability

Data are contained within the article and Supplementary Materials.

## Supplementary Materials

The following supporting information can be downloaded at: https://www.mdpi.com/article/10.3390/molecules29112513/s1, such as raw data, Quality assessment (QC) and quality control (QA), Normalization and significantly different substances.

## References

[1] Gunde-Cimerman N., Plemenitaš A., Oren A. (2018). Strategies of Adaptation of Microorganisms of the Three Domains of Life to High Salt Concentrations. FEMS Microbiol. Rev..

[2] Kogej T., Stein M., Volkmann M., Gorbushina A.A., Galinski E.A., Gunde-Cimerman N. (2007). Osmotic Adaptation of the Halophilic Fungus Hortaea Werneckii: Role of Osmolytes and Melanization. Microbiology.

[3] Chen D.-D., Fang B.-Z., Manzoor A., Liu Y.-H., Li L., Mohamad O.A.A., Shu W.-S., Li W.-J. (2021). Revealing the Salinity Adaptation Mechanism in Halotolerant Bacterium Egicoccus Halophilus EGI 80432T by Physiological Analysis and Comparative Transcriptomics. Appl. Microbiol. Biotechnol..

[4] Jiménez-Gómez I., Valdés-Muñoz G., Moreno-Ulloa A., Pérez-Llano Y., Moreno-Perlín T., Silva-Jiménez H., Barreto-Curiel F., del Sánchez-Carbente M.R., Folch-Mallol J.L., Gunde-Cimerman N. (2022). Surviving in the Brine: A Multi-Omics Approach for Understanding the Physiology of the Halophile Fungus Aspergillus Sydowii at Saturated NaCl Concentration. Front. Microbiol..

[5] Beever R.E., Laracy E.P. (1986). Osmotic Adjustment in the Filamentous Fungus Aspergillus Nidulans. J. Bacteriol..

[6] Jiménez-Gómez I., Valdés-Muñoz G., Moreno-Perlin T., Mouriño-Pérez R.R., del Sánchez-Carbente M.R., Folch-Mallol J.L., Pérez-Llano Y., Gunde-Cimerman N., del Sánchez N.C., Batista-García R.A. (2020). Haloadaptative Responses of Aspergillus Sydowii to Extreme Water Deprivation: Morphology, Compatible Solutes, and Oxidative Stress at NaCl Saturation. J. Fungi.

[7] Ding X., Liu K., Lu Y., Gong G. (2019). Morphological, Transcriptional, and Metabolic Analyses of Osmotic-Adapted Mechanisms of the Halophilic Aspergillus Montevidensis ZYD4 under Hypersaline Conditions. Appl. Microbiol. Biotechnol..

[8] Kis-Papo T., Weig A.R., Riley R., Peršoh D., Salamov A., Sun H., Lipzen A., Wasser S.P., Rambold G., Grigoriev I.V. (2014). Genomic Adaptations of the Halophilic Dead Sea Filamentous Fungus Eurotium Rubrum. Nat. Commun..

[9] Tafer H., Poyntner C., Lopandic K., Sterflinger K., Piñar G. (2019). Back to the Salt Mines: Genome and Transcriptome Comparisons of the Halophilic Fungus Aspergillus Salisburgensis and Its Halotolerant Relative Aspergillus Sclerotialis. Genes.

[10] Kogej T., Ramos J., Plemenitaš A., Gunde-Cimerman N. (2005). The Halophilic Fungus *Hortaea Werneckii* and the Halotolerant Fungus *Aureobasidium Pullulans* Maintain Low Intracellular Cation Concentrations in Hypersaline Environments. Appl. Environ. Microbiol..

[11] Plemenitaš A., Lenassi M., Konte T., Kejžar A., Zajc J., Gostinčar C., Gunde-Cimerman N. (2014). Adaptation to High Salt Concentrations in Halotolerant/Halophilic Fungi: A Molecular Perspective. Front. Microbiol..

[12] Henry C., Bledsoe S.W., Griffiths C.A., Kollman A., Paul M.J., Sakr S., Lagrimini L.M. (2015). Differential Role for Trehalose Metabolism in Salt-Stressed Maize. Plant Physiol..

[13] Gostinčar C., Lenassi M., Gunde-Cimerman N., Plemenitaš A. (2011). Fungal Adaptation to Extremely High Salt Concentrations. Advances in Applied Microbiology.

[14] Kralj Kunčič M., Kogej T., Drobne D., Gunde-Cimerman N. (2010). Morphological Response of the Halophilic Fungal Genus *Wallemia* to High Salinity. Appl. Environ. Microbiol..

[15] Pérez-Llano Y., Rodríguez-Pupo E.C., Druzhinina I.S., Chenthamara K., Cai F., Gunde-Cimerman N., Zalar P., Gostinčar C., Kostanjšek R., Folch-Mallol J.L. (2020). Stress Reshapes the Physiological Response of Halophile Fungi to Salinity. Cells.

[16] Rodríguez-Pupo E.C., Pérez-Llano Y., Tinoco-Valencia J.R., Sánchez N.S., Padilla-Garfias F., Calahorra M., del Sánchez N.C., Sánchez-Reyes A., del Rodríguez-Hernández M.R., Peña A. (2021). Osmolyte Signatures for the Protection of Aspergillus Sydowii Cells under Halophilic Conditions and Osmotic Shock. J. Fungi.

[17] Ge Y., Wang Y., Liu Y., Tan Y., Ren X., Zhang X., Hyde K.D., Liu Y., Liu Z. (2016). Comparative Genomic and Transcriptomic Analyses of the Fuzhuan Brick Tea-Fermentation Fungus Aspergillus Cristatus. BMC Genom..

[18] Ge Y., Yu F., Tan Y., Zhang X., Liu Z. (2017). Comparative Transcriptome Sequence Analysis of Sporulation-Related Genes of Aspergillus Cristatus in Response to Low and High Osmolarity. Curr. Microbiol..

[19] Liu K.-H., Ding X.-W., Narsing Rao M.P., Zhang B., Zhang Y.-G., Liu F.-H., Liu B.-B., Xiao M., Li W.-J. (2017). Morphological and Transcriptomic Analysis Reveals the Osmoadaptive Response of Endophytic Fungus Aspergillus Montevidensis ZYD4 to High Salt Stress. Front. Microbiol..

[20] Xiao Y., Li M., Liu Y., Xu S., Zhong K., Wu Y., Gao H. (2021). The Effect of Eurotium Cristatum (MF800948) Fermentation on the Quality of Autumn Green Tea. Food Chem..

[21] Efimenko T.A., Shanenko E.F., Mukhamedzhanova T.G., Efremenkova O.V., Nikolayev Y.A., Bilanenko E.N., Gernet M.V., Grishin A.G., Serykh I.N., Shevelev S.V. (2021). Eurotium Cristatum Postfermentation of Fireweed and Apple Tree Leaf Herbal Teas. Int. J. Food Sci..

[22] Xiao Y., He C., Chen Y., Ho C.-T., Wu X., Huang Y., Gao Y., Hou A., Li Z., Wang Y. (2022). UPLC–QQQ–MS/MS-Based Widely Targeted Metabolomic Analysis Reveals the Effect of Solid-State Fermentation with Eurotium Cristatum on the Dynamic Changes in the Metabolite Profile of Dark Tea. Food Chem..

[23] Xiao Y., Zhong K., Bai J., Wu Y., Gao H. (2020). Insight into Effects of Isolated *Eurotium Cristatum* from Pingwu Fuzhuan Brick Tea on the Fermentation Process and Quality Characteristics of Fuzhuan Brick Tea. J. Sci. Food Agric..

[24] van Iersel M.L.P.S., Cnubben N.H.P., Smink N., Koeman J.H., van Bladeren P.J. (1999). Interactions of Prostaglandin A2 with the Glutathione-Mediated Biotransformation System. Biochem. Pharmacol..

[25] Warris A., Ballou E.R. (2019). Oxidative Responses and Fungal Infection Biology. Semin. Cell Dev. Biol..

[26] Miller G., Suzuki N., Ciftci-Yilmaz S., Mittler R. (2010). Reactive Oxygen Species Homeostasis and Signalling during Drought and Salinity Stresses. Plant Cell Environ..

[27] Kumar S., Kalyanasundaram G.T., Gummadi S.N. (2011). Differential Response of the Catalase, Superoxide Dismutase and Glycerol-3-Phosphate Dehydrogenase to Different Environmental Stresses in Debaryomyces Nepalensis NCYC 3413. Curr. Microbiol..

[28] Ren X., Bao Y., Zhu Y., Liu S., Peng Z., Zhang Y., Zhou G. (2019). Isorhamnetin, Hispidulin, and Cirsimaritin Identified in Tamarix Ramosissima Barks from Southern Xinjiang and Their Antioxidant and Antimicrobial Activities. Molecules.

[29] Liu K., Zhao F., Yan J., Xia Z., Jiang D., Ma P. (2020). Hispidulin: A Promising Flavonoid with Diverse Anti-Cancer Properties. Life Sci..

[30] Stompor M. (2020). A Review on Sources and Pharmacological Aspects of Sakuranetin. Nutrients.

[31] Li J., Wang T., Liu P., Yang F., Wang X., Zheng W., Sun W. (2021). Hesperetin Ameliorates Hepatic Oxidative Stress and Inflammation *via* the PI3K/AKT-Nrf2-ARE Pathway in Oleic Acid-Induced HepG2 Cells and a Rat Model of High-Fat Diet-Induced NAFLD. Food Funct..

[32] Abarikwu S.O., Njoku R.-C.C., John I.G., Amadi B.A., Mgbudom-Okah C.J., Onuah C.L. (2022). Antioxidant and Anti-Inflammatory Protective Effects of Rutin and Kolaviron against Busulfan-Induced Testicular Injuries in Rats. Syst. Biol. Reprod. Med..

[33] Shen T., Li H.-Z., Li A.-L., Li Y.-R., Wang X.-N., Ren D.-M. (2018). Homoeriodictyol Protects Human Endothelial Cells against Oxidative Insults through Activation of Nrf2 and Inhibition of Mitochondrial Dysfunction. Vascul Pharmacol..

[34] Slyshenkov V.S., Dymkowska D., Wojtczak L. (2004). Pantothenic Acid and Pantothenol Increase Biosynthesis of Glutathione by Boosting Cell Energetics. FEBS Lett..

[35] Morell S., Follmann H., De Tullio M., Häberlein I. (1997). Dehydroascorbate and Dehydroascorbate Reductase Are Phantom Indicators of Oxidative Stress in Plants. FEBS Lett..

[36] Oren A. (1999). Bioenergetic Aspects of Halophilism. Microbiol. Mol. Biol. Rev..

[37] Peng J., Zhang D., Ma Y., Wang G., Guo Z., Lu J. (2014). Protective Effect of Fluvastatin on Influenza Virus Infection. Mol. Med. Rep..

[38] Tavakkoli A., Johnston T.P., Sahebkar A. (2020). Antifungal Effects of Statins. Pharmacol. Ther..

[39] Xiao X., Zhou L., Ma X. (2011). Effect of Adding Perilla Leaf to Monascus Fermentation on Its Antioxidant Activity. China Brewing..

[40] Wang B.S., Li B.S., Zeng Q.X., Liu H.X. (2008). Antioxidant and Free Radical Scavenging Activities of Pigments Extracted from Molasses Alcohol Wastewater. Food Chem..

[41] Wu S., Zhang J., Chen Y., Zhong L. (2019). Screening and Identification of Lactic Acid Bacteria Strains with Antioxidant activities in Fermented Food. J. Zhejiang Univ. Technol..

