# Supplementary material for: Material Composition Characteristics of Aspergillus cristatus under High Salt Stress through LC–MS Metabolomics
Source: Molecules. 2024 May 26;29(11):2513. doi: 10.3390/molecules29112513 (PMC11173666; doi:10.3390/molecules29112513)
Supplement: Supplementary file 1 [file molecules-29-02513-s001.zip › Supporting Figure.pdf]

1. Quality assessment (QC) and quality control (QA)

In the process of data quality evaluation, the distribution of PC1 values at all sample points can be used to evaluate whether the laboratory sample preparation and sample measurement processes are in a controllable state. Sample points exceeding the control limit (3 times standard deviation) are considered outliers. As shown in Figure S1, generally speaking, all points will be within the control boundary. Among them, most points will fluctuate up and down around the mean axis within 2 times the standard deviation, and a small number of points will approach the control limit.

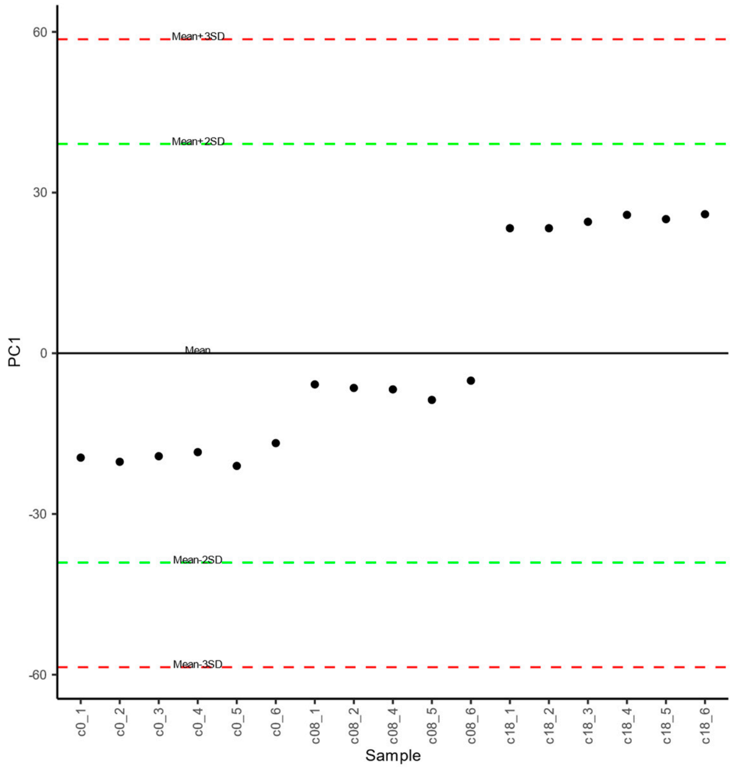

Figure S1. All Sample_PC1.

Quality control is one of the fundamental concepts in biological analysis, used to ensure the repeatability and accuracy of omics data. Due to the direct contact between the chromatographic system and mass spectrometry with the sample, as the number of analyzed samples increases, the chromatographic column and mass spectrometry will gradually become contaminated, leading to signal drift and causing systematic measurement errors. The behavior of tracking the entire data collection process by repeatedly using the same quality control sample (QC sample) has been recommended and used by most experts in the field of analytical chemistry. Quality control samples are used to evaluate the signal drift of the entire mass spectrometry data during the collection process, which can be further identified, corrected, and improved by precise algorithms to enhance the quality of the data. This process uses the QC-RFSC algorithm

of the StatTarget package in R language to correct the signal peaks of each sample's features (each metabolite), and records the correction effect of each metabolite. QC samples are samples obtained by mixing equal amounts of all samples. During the signal data collection process, QC samples are inserted at the beginning, end, and middle positions to record signal drift. All QC samples are the same. If there is no signal drift, the signal strength of QC samples should remain unchanged during the data acquisition process. As shown in Figure S2- Figure S6, after correcting the signal drift, if the QC sample points gather together in the PCA plot, it proves that the correction effect is good.

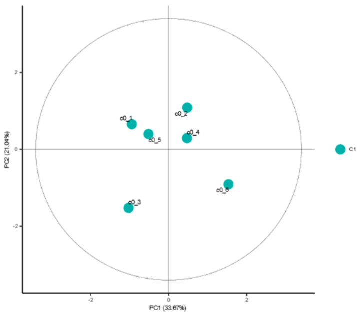

Figure S2. C1_PCA_QA.

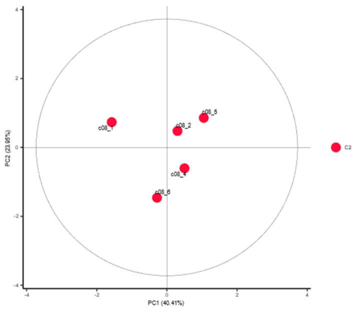

Figure S3. C2_PCA_QA.

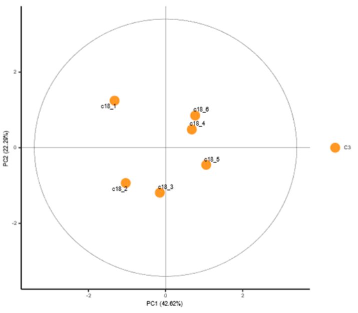

Figure S4. C3_PCA_QA.

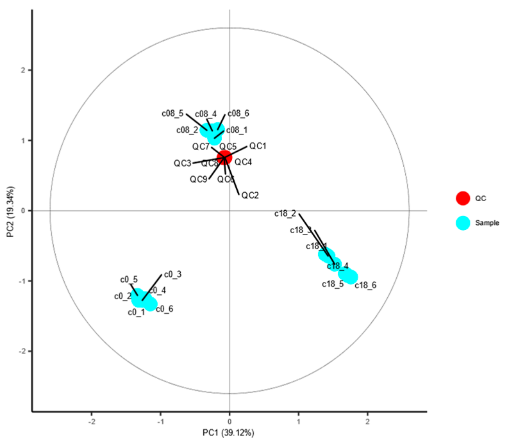

Figure S5. neg _PCA_QC.

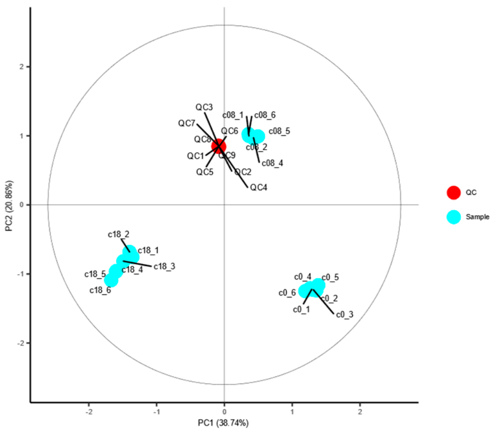

Figure S6. pos _PCA_QC.

2. Normalization

Within sample correction: dividing the abundance of all features in the sample by the median abundance of the sample (similar to relative abundance calculation); This is to correct the library size. During the measurement process, there is a significant difference in the total metabolite content obtained from each sample, which is usually a systematic error caused by the sample collection and measurement process. Dividing by the median, mean, or total is a common way to correct this systematic error.

Content matrix correction: log conversion is performed on all content values; T-test and ANOVA methods for comparing differences require that metabolite content follows a normal distribution. Therefore, we generally use log transformation to make the distribution of metabolite content close to a normal distribution.

Feature internal correction: subtracting the mean abundance of all samples corresponding to the feature and then dividing it by the standard deviation of the feature abundance; The purpose of feature internal correction is to ensure that the mean and standard deviation (or median, quartile, scale) of all metabolites are at the same level; Analysis such as PCA, PLSDA, OPLSDA, and machine learning, if metabolite standardization is not carried out, the importance of metabolites with high mean and standard deviation will tend to be higher than that of metabolites with low mean and standard deviation. Such results are obviously not what we want, and only those with large differences between groups should have high importance.

As shown in Figures S7 and Figures S8, before standardization correction, the median and upper and lower quartiles of metabolite content varied greatly, but after standardization correction, they were basically at the same level.

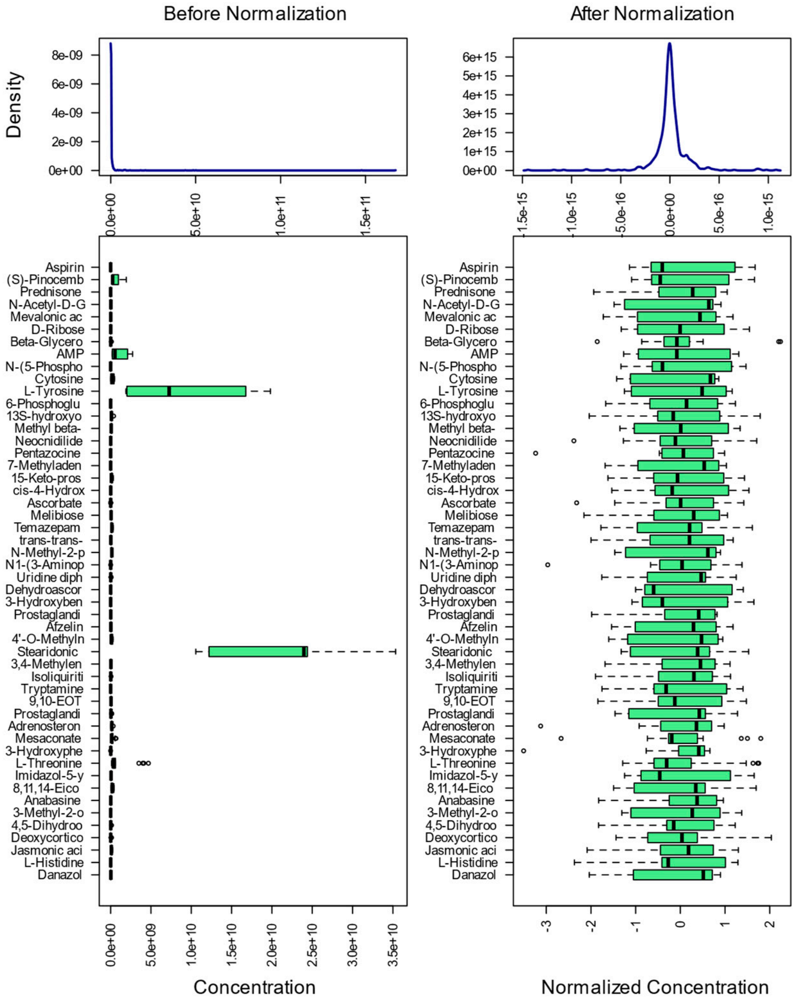

Figure S7. compound wise normalization.

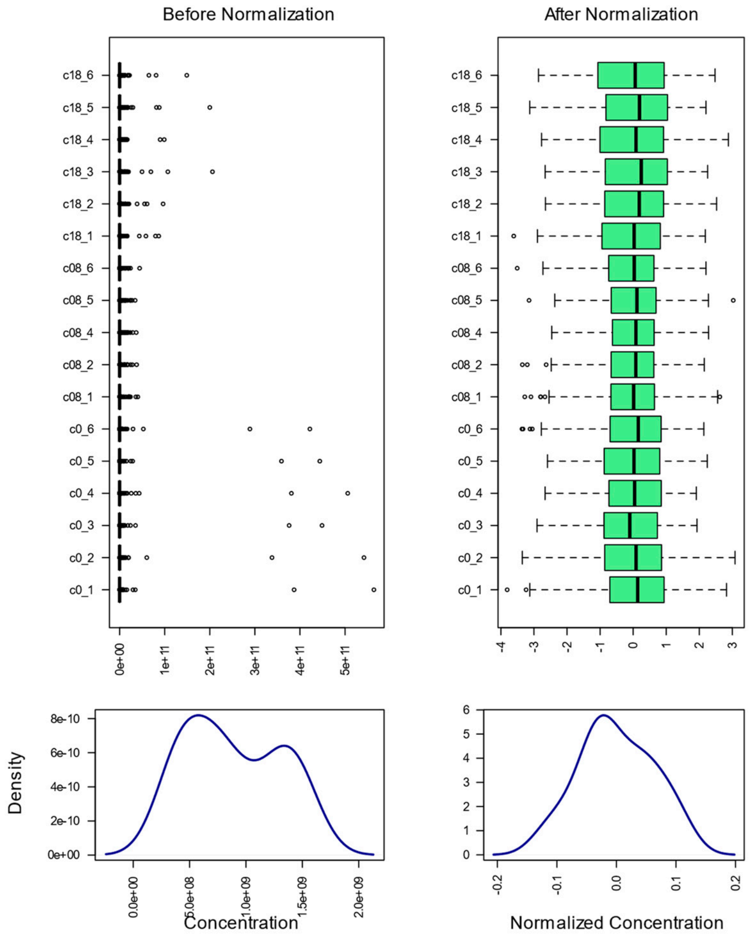

Figure S8. sample wise normalization.
